# *Toxoplasma gondii* in wild and domestic animals from New Caledonia

**DOI:** 10.1051/parasite/2011184345

**Published:** 2011-11-15

**Authors:** C. Roqueplo, L. Halos, O. Cabre, B. Davoust

**Affiliations:** 1 Groupe de travail en épidémiologie animale du Service de Santé des Armées Marseille France; 2 ANSES, ENVA, UPEC-UMR BIPAR, École nationale vétérinaire d’Alfort Maisons-Alfort France; 3 Direction interarmées du Service de Santé des Forces Armées en Nouvelle-Calédonie Nouméa Nouvelle-Calédonie

**Keywords:** *Toxoplasma gondii*, New Caledonia, Rusa deer, livestock, horse, domestic carnivore, *Toxoplasma gondii*, Nouvelle-Calédonie, cerf rusa, animal d’élevage, cheval, carnivore domestique

## Abstract

Samples (serum or meat juice) collected from 205 animals in New Caledonia in April 2009 were tested for antibodies against *Toxoplasma gondii* by ELISA using the multi-species ID Screen^®^ Toxoplasmosis Indirect kit (IDVET, Montpellier). Antibodies to *T. gondii* were detected in 2% (1/49) of the pigs, in 3.3% (1/30) of the cattle, in 13.8% (4/29) of Rusa deers, in 16% (4/25) of the horses, in 32.8% (21/64) of the dogs, and in 50% (4/8) of cats. Statistically, no significant difference was observed between *T. gondii* seroprevalence and age or sex. No survey on the prevalence of *T. gondii* in animals has ever been conducted in New Caledonia and this is the first serological evidence of *T. gondii* in Rusa deer (*Cervus timorensis russa*). These results indicate an important circulation of *T. gondii* exists in the animal populations of New Caledonia. In view of humans being exposed, it is advisable to insist on sanitary education and on respect for good hygienic and food practice.

## Abstract (fr)

205 échantillons de deux types (sang ou muscle) ont été recueillis en avril 2009 en Nouvelle-Calédonie. Une recherche sérologique sur sérum et sur fluide musculaire a été menée par la technique ELISA à l’aide du kit multi-espèce ID Screen^®^ Toxoplasmosis Indirect (IDVET, Montpellier) pour la détection d’anticorps dirigés contre la protéine P30 de *Toxoplasma gondii*. Les résultats montrent une séropositivité de 2 % (1/49) chez les porcs, 3,3 % (1/30) chez les bovins, 13,8 % (4/29) chez les cerfs, 16 % (4/25) chez les chevaux, 32,8 % (21/64) chez les chiens et 50 % (4/8) chez les chats. D’après nos résultats, ni le sexe, ni l’âge de l’animal n’interviennent de façon statistiquement significative sur sa positivité vis-à-vis de *T. gondii*. À notre connaissance, c’est la première fois qu’une enquête sérologique est menée sur des animaux en Nouvelle-Calédonie, et la première mise en évidence sérologique du parasite chez le cerf rusa (*Cervus timorensis russa*). Ces résultats sérologiques indiquent une circulation importante de *T. gondii* dans les populations animales de Nouvelle-Calédonie. L’homme étant particulièrement exposé, il convient, tout particulièrement, d’insister sur l’éducation sanitaire et sur le respect des bonnes pratiques hygiéniques et alimentaires.

Toxoplasmosis, the most common parasitic zoonosis worldwide, is caused by the intracellular protozoan *Toxoplasma gondii*. The infection is usually asymptomatic in immunocompetent patients. Severe forms are above all observed in cases of congenital transmission and in immunosuppressed patients. Cerebral toxoplasmosis represented the index diagnosis of AIDS in approximately 12 % of cases in France in 2009, but it can also be an evolutionary complication. In New Caledonia in 2009, 17 % of the patients infected by HIV presented simultaneously or successively with cerebral toxoplasmosis (DASS-NC, 2009). It is an important public health problem as, between 1986 and 2009, 344 HIV-positive patients were detected in New Caledonia, among which 123 developed AIDS (Cazein *et al.*, 2010; DASS-NC, 2009). A study performed in 2000 among 2,416 women of childbearing age showed a prevalence of *Toxoplasma* antibodies in 56.7 %, which is higher than the prevalence of 43.8 % reported in the metropolitan area (Breurec *et al.*, 2004).

Cats and wild felidae play a major role in the epidemiology of *T. gondii*, as they are the only known definitive hosts that shed environmentally resistant oocysts in their faeces. The domestic cat (*Felis silvestris catus*) was introduced in New Caledonia, initially on the Great Land, at the beginning of the 1840’s by European navigators who brought them in to fight rats (Gargominy *et al.*, 1996). The implantation of cats was successful as they can now be found in every environment, even in sites located far from human habitats (top of the Koniambo) and in natural parks in the Southern county (Rouys & Theueukauf, 2003). These wandering cats live freely and may, after a primo-infection with *T. gondii*, spread a large number of oocysts in the environment and contaminate grazing zones for cattle (Tenter *et al.*, 2000).

In New Caledonia, the consumption of deer meat (home-made delicatessen, carpaccio…) is common amongst residents. Cervids have been considered a source of *T. gondii* infection in humans worldwide. Consumption of inadequately cooked or raw meat from infected cervids has reportedly caused acute and ocular toxoplasmosis in deer hunters in the United States (Ross *et al.*, 2001). Evisceration and handling of game may also represent a risk for human infection (Dubey, 1994). The delicatessen and the smoked meats can also be sources of toxoplasmosis. Aspinall (2002) showed the presence of *T. gondii* DNA in 27/71 (38 %) tested samples (sausages, smoked ham…). *Cervus timorensis russa* was introduced to New Caledonia in 1862 from Indonesia (Java) and New Caledonia has one of the most important populations of Rusa deer in the world. The population of Rusa deer has grown over the last decades (Gargominy *et al.*, 1996) and the island is a privileged destination for hunting in mid-tropics. The deer live free in the savannah or in semi freedom in big breeding farms on immense plots of land.

Numerous studies have been performed in many countries on the prevalence of *T. gondii* in meat-producing animals, but the distribution of *T. gondii* within animal populations has never been the object of studies in New Caledonia. Public health concerns associated with *T. gondii* clearly indicate the need for epidemiological investigations of this infection in animals, particularly those that can be used as a source of food. The aim of the present study was to estimate the prevalence of *T. gondii* infection among meat-producing and other animals in New Caledonia.

## Materials and Methods

A total of 205 samples of blood or muscle were collected in April 2009 in New Caledonia. Sera were taken from 30 cattle, 29 deer (*Cervus timorensis russa*), 25 horses, 64 dogs and 8 cats, while 49 samples of pig diaphragm and masseter were obtained from random carcasses in a commercial slaughterhouse.

The sera from cattle and deer and muscular fluids from pigs were collected by veterinarians in a slaughterhouse of the Office of marketing and refrigerated storage (OCEF) of Bourail. Dog sera were collected from several sites. Some lived in kennels, either in the municipal pound of Nouméa, or with the Society for the Prevention of Cruelty to Animals (SPCA) (Koutio, Dumbea). Other dogs belonged to the military dog squads of the naval air station Tontouta, and of the Pacific marine infantry regiment (detachment of the Nandail’s station). Cat sera were collected in the municipal pound of Nouméa. Horse sera came partly from stables of Païta, and the others from the Népoui police squad. The samples of meat (100 g) were frozen according to the protocol of the French Agency of sanitary security of food (ANSES) before being sent to the National reference laboratory for foodborne parasites in Maisons-Alfort (Halos *et al.*, 2010). Meat juice samples from pigs were prepared from diaphragm and masseter tissue. After centrifugation, the blood samples were stored at - 20 °C prior to dispatch. An ELISA test was performed on serum and on muscular fluid, using a commercial test kit (kit multi-species ID Screen® Toxoplasmosis Indirect, IDVET, Montpellier) for the detection of antibodies against the *Toxoplasma* P30 protein. The muscular fluids represent a very interesting alternative matrix for serological analyses, because they are simpler to collect. However, because of a weaker antibody concentration in these fluids than in the serum, the muscular fluid was diluted at 1/2 and not 1/10 (Nöckler *et al.*, 2005).

As there are no standardized validated serologic tests for correlating seropositivity with the presence of infectious parasites in meat, the diagnostic accuracy of different serological methods is usually assessed by comparing their results with those obtained by other serological tests, such as the modified agglutination test (MAT) (Werre, 2002). A previous study has shown a high degree of agreement between the MAT and ELISA tests, performed on meat fluids (Halos *et al.*, 2010), allowing the use of ELISA in seroprevalence studies.

The results were analysed by 2 X K contingency tables of exposure variables. The outcome variable was positivity to *T. gondii* (ELISA) and the independent variables were: age, sex and geographical areas.

Odds ratios (OR), 95 % confidence intervals (CI) and *p* values were calculated separately for each variable using the Epi Info Software (v5.01, CDC Atlanta, USA). The Chi-square, or the Fisher exact test test if appropriate, was used to evaluate associations (α = 5 %). Differences were considered statistically significant when p ≤ 0.05.

Cattle, horse, deer and dogs were classified into age groups, which differed according to species. However the low numbers examined and positive animals in some species determined the low number of age groups formed to allow for statistical analysis. Accordingly, deer, horses and cattle were all classified into two age groups, young and adults, which were defined as follows: deer, ≤ 2 year old, n = 19, and > 2 year old, n = 1; horses, ≤ 6 year old, n = 11, and > 6 year old, n = 14; cattle, < 4 year old, n = 17, and ≥ 4 year old, n = 13. Dogs were classified into three age groups: young (≤ 2 years of age, n = 15), adult (between 2-8 year old, n = 34) and senior (≥ 8 year old, n = 15).

## Results

Table I shows the characteristics of the populations, and summarises the results of serology. The prevalence of toxoplasmosis among cattle, horse, deer, dog, cat and pig was 3.3 %, 16 %, 13.8 %, 32.8 %, 50 % and 2 %, respectively.

**Table I.** Distribution of the study population according to age and sex and prevalence of *Toxoplasma gondii*.

Specie	Number	Seroprevalence %	CI_95%_	Sex ratio (Male) %	Age in years (mean ± sd)	Age range (min - max)
Cattle	30	3.3 (1/30)	0.08 – 17.2	65	3.0 ± 2.3	0.5 – 11.0
Horse	25	16.0 (4/25)	4.5 – 36	76	8.0 ± 4.7	2.0 – 23.0
Deer	29	13.8 (4/29)	3.9 – 31.6	69	2.7 ± 1.1	1.5 – 4.5
Dog	64	32.8 (21/64)	21.6 – 45.7	65	4.3 ± 2.8	0.25 – 13.0
Cat	8	50.0 (4/8)	15.7 – 84.3	NA	NA	NA
Pig^1^	49	2.0 (1/49)	0.005 – 10.8	NA	NA	NA

1 results obtained on meat juice.

According to the analyzed variables, a single difference in the seroprevalence was found, this being between dogs living in kennels in SPCA (38.7 %, 19/49) and military dogs (6.6 %, 1/15) (p = 0.01).

No statistically significant difference in the seroprevalence of *T. gondii* was observed according to sex and age in cattle (p = 0.6; p = 0.5), horses (p = 0.3; p = 0.1), deer (p = 0.3; p = 0.5), and dogs (p = 0.5; 0.6). In addition, there was no statistically significant difference (p = 0.1) in the seroprevalence in horses from Païta and Nepoui areas.

## Discussion

Reports of antibodies to *T. gondii* in cervids originate mainly from the USA and Europe, (Vanek *et al.*, 1996; Dubey *et al.*, 2004, Gauss *et al.*, 2006) but, to our knowledge, the present study is the first report of seroprevalence of *T. gondii* in Rusa deer (*Cervus timorensis russa*). Whilst the 13.8 % seroprevalence of *T. gondii* in Rusa deer was not related to age and sex, this is probably due to the too low number of animals analyzed.

The prevalence observed in deer (13.8 %, 4/29) is not significantly different (p = 0.15) from that observed in the cattle (3.3 %, 1/30), which live in the same breeding conditions.

Cattle are generally described as insensitive to *T. gondii* infection. Cattle would harbour few parasite tissue cysts, which may not persist for the lifetime of the host (Esteban-Redondo *et al.*, 1999; Dubey & Thulliez, 1993; Dubey *et al.*, 1985).

The seroprevalence observed in dogs of this study (32.8 %) is comparable to that observed in studies in metropolitan France (38.5 %, 1,378/3,580; Cabannes *et al.*, 1998). The difference in the prevalence between dogs living in kennels in SPCA and military dogs may be explained by the conditions of life of stray dogs which are favourable to contraction of the parasite (contact with felids, consumption of tissue cysts in infected animal tissues…).

Numerous studies have been performed in many countries on the prevalence of *T. gondii* in cats. Although in our study the number of cat samples (n = 8) was small, this high prevalence is relatively close to the prevalences of 43.1 % (218/506) and of 40 % (10/25), observed in surveys of wandering cats in metropolitan France and Dakar (Senegal), respectively (Cabannes *et al.*, 1998). Outdoor life favours ingestion of *T. gondii* cysts by means of their intermediate consumption by rodents and birds, or by the consumption of remnants of human meals containing meat or internal organs of cattle or game. Therefore, cats are constantly exposed to the infection. The seropositive cats had probably previously excreted oocysts and contaminated the environment in New Caledonia.

In our study the prevalence in pigs was 2 %. Prevalence of *T. gondii* in pigs varies, but generally exceeds 10 % in most countries, for instance 26.83 % of domestic pigs in Zimbabwe (Hove *et al.*, 2005), and 39 % of domestic pigs in Ghana (Arko-Mensah *et al.*, 2000). Pigs may become infected by ingesting feed or water contaminated with cat faeces, by cannibalism of other infected dead pigs, by ear and tail biting or by eating infected rodents or other uncooked meat.

The impact of breeding system on animal contamination is well known (Hove *et al.*, 2005). In New Caledonia, pig farms, which are mainly located in the southern county (87 %) are semi-intensive, with a high-tech level that could be compared to the European mid-level (DAVAR, 2009).

In our study the prevalence in horses was 16 %. Similar results were reported from investigations in horses from Argentina (13 %), and Brazil (16 %) (Dubey *et al.*, 1999a, b). Horses can obviously become infected, and caution should be paid to the consumption of undercooked horsemeat which may contain the parasite. *T. gondii* tissue cysts in the muscles of meat-producing animals, which occur most frequently in pig tissues, less frequently in deer and horses and rarely in beef, are an important source of human infection. The public health risk of contracting toxoplasmosis during consumption of meat is real.

## Conclusion

Our serological results indicate an important circulation of *T. gondii* in the animal populations of New Caledonia, in particular in domestic animals used for human consumption. Although further studies in New Caledonia are needed to provide more information on this issue, the presented results show a clear public health risk, warranting improvement of the sanitary education and respect for good hygienic and food practices.
